# The Impact of Prior Mammograms on the Diagnostic Performance of Radiologists in Early Breast Cancer Detection: A Focus on Breast Density, Lesion Features and Vendors Using Wholly Digital Screening Cases

**DOI:** 10.3390/cancers15041339

**Published:** 2023-02-20

**Authors:** Phuong Dung (Yun) Trieu, Natacha Borecky, Tong Li, Patrick C. Brennan, Melissa L. Barron, Sarah J. Lewis

**Affiliations:** 1 Department of Clinical Imaging, Faculty of Medicine and Health, The University of Sydney, Level 7-D18, Susan Wakil Health Building, Camperdown, NSW 2006, Australia; natacha.borecky@sydney.edu.au (N.B.); t.li@sydney.edu.au (T.L.); patrick.brennan@sydney.edu.au (P.C.B.); melissa.robinson@sydney.edu.au (M.L.B.); sarah.lewis@sydney.edu.au (S.J.L.); 2 BreastScreen New South Wales (North Coast), Lismore, NSW P.O. Box 1098, Australia

**Keywords:** breast cancer, screening, radiology, previous images

## Abstract

**Simple Summary:**

This study explored the diagnostic efficacy of radiologists when reading screening mammograms in the absence of previous images (NP), and with prior images obtained from the same (SP) and different vendors (DP). There were 612 radiologists reading 9 mammogram test sets (361-normal and 179-cancer) with 245 cases having prior images from the same vendor and 129 from different vendors. Radiologists obtained 12.8% and 10.3% higher sensitivity in NP and DP than SP. The ROC AUC for NP and DP were also significantly higher than SP. The odds ratio of true positive for NP and DP was 1.6 and 1.5, respectively, relative to SP cases. Radiologists were more likely to detect architectural distortion (OR = 3.2) and calcifications (OR = 2.85) in DP than SP. The findings suggest exploring a mixed reading strategy in viewing cases with prior mammograms acquired from the same and different manufacturers to enhance the diagnostic accuracy in the digital era.

**Abstract:**

Background: This study aims to investigate the diagnostic efficacy of radiologists when reading screening mammograms in the absence of previous images, and with the presence of prior images from the same and different vendors. Methods: 612 radiologists’ readings across 9 test sets, consisting of 540 screening mammograms (361-normal and 179-cancer) with 245 cases having prior images obtained from same vendor as current images, 129 from a different vendor and 166 cases having no prior images, were retrospectively analysed. True positive (sensitivity), true negative (specificity) and area under ROC curve (AUC) values of radiologists were calculated for three groups of cases (without prior images (NP), with prior images from same vendor (SP), and with prior images from different vendor (DP)). Logistic regression was used to estimate the odds ratio (OR) of true positive, true negative and true cancer localization among case groups with different levels of breast density and lesion characteristics. Results: Radiologists obtained 12.8% and 10.3% higher sensitivity in NP and DP than SP (0.803-and-0.785 vs. 0.712; *p* < 0.0001). Specificity in NP and DP cases were 4.8% and 2.0% lower than SP cases (0.749 and 0.771 vs. 0.787). The AUC values for NP and DP were significantly higher than SP cases across different levels of breast density (0.814-and-0.820 vs. 0.782; *p* < 0.0001). The odds ratio (OR) of true positive for NP relative to SP was 1.6 (*p* < 0.0001) and DP relative to SP was 1.5 (*p* < 0.0001). Radiologists were more like to detect architectural distortion in DP than SP cases (OR = 3.2; *p* < 0.0001), whilst the OR for abnormal calcifications was 2.85 (*p* < 0.0001). Conclusions: Cases without previous mammograms or with prior mammograms obtained from different vendors were more likely to benefit radiologists in cancer detection, whilst prior mammograms undertaken from the same vendor were more useful for radiologists in evaluating normal cases.

## 1. Background

In breast cancer screening, treatment outcomes are reliant on accurate interpretation by radiologists or other verified readers for the early detection of abnormal lesions on breast images. Digital mammography, with high specificity and sensitivity (over 80%) [1], is the main imaging approach employed for breast cancer diagnosis and screening programs worldwide. A mammogram is used to assess abnormal lesions and is suitable for screening for breast cancer in women who have no signs or symptoms. In Australia, where a national breast screening program has been implemented since 1991, women between the ages of 50 and 74 are invited for mammography examinations biannually [2].

Breast screening radiologists are often required to read high volumes of mammograms in order to increase their accuracy in identifying malignant findings [3], as mammography is considered a challenging visual task due to the superimposition of abnormal findings on normal breast tissues [4]. Consequently, an effective workflow in the reading process is essential. With the transition in technology from film-screen (FS) to full-field digital mammography (FFDM) in recent decades, traditional film image viewing was replaced with softcopy image reading equipment, often enabling the radiologist to conveniently access available images, including previous mammograms, to compare with current images. Previous studies suggested that viewing prior mammograms could help to decrease recall rate but may not be as beneficial in the detection of abnormal lesions; however, these studies used the older FS technology or had a low number of participants (with one to three radiologists involved in the studies) [5,6]. In comparing FS and digital mammograms, it is reported that digital mammography produces higher quality images through digital sensors that increase resolution and clarity, which leads to higher cancer detection than with FS [7] Radiographers and radiologists can also enhance, magnify or adjust contrasts of digital mammograms more easily than with FS mammograms, allowing for different viewing parameters around areas of interest [8]. In addition, the influence of reading experiences [9], and breast density that might impact on the rates of missed breast cancer due to the masking effect of dense breast tissue and overlapping structures [4] were not clarified in the previous research. Furthermore, the impact of the vendor origin for the current and prior cases has not been explored in the former studies. Therefore, this study aims to investigate the diagnostic performances of radiologists in cases without prior images and cases with prior images acquired from the same and different manufacturers to understand in which situations prior mammogram availability might influence the diagnostic accuracy of radiologists.

## 2. Methods

### 2.1. Oversight

This study was conducted with data retrospectively collected from the BREAST (Breastscreen REader Assessment STrategy) program. BREAST is an educational and training platform for continuous professional development of BreastScreen readers in Australia [10,11]. Ethical approval was obtained from the Research Ethics Institution Committee of the University of Sydney (2019/013). The informed consent was sought from each participant for the data collection.

### 2.2. Study Population

Data of 612 Australian radiologists’ readings across 9 mammography cancer-enriched test sets, with 60 cases per set, were collected between September 2014 and November 2021. Only the first-time readings of each test set by each radiologist were collected for this study so as not to introduce a memory bias. The participants self-reported demographic details in terms of the number of years of experience and the number of cases of reading per week. A total of 43% of radiologists had equal to or greater than 10 years of experience reading mammograms and 39.6% of radiologists read more than 100 cases per week, which is equal to 4800 mammographic readings per year. The details of participants are summarized in Table 1.

### 2.3. Mammogram Collection

**Figure 1:**
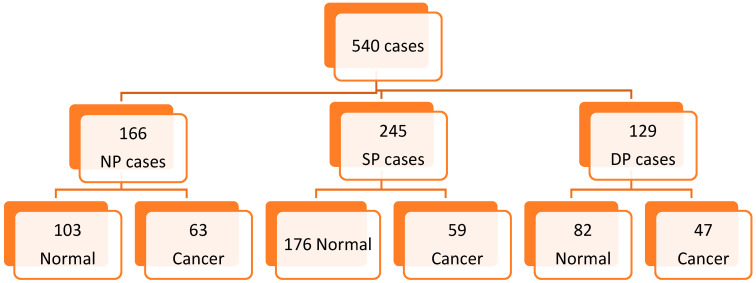
Case distribution among different categories (NP—without prior images, SP—with prior images from same vendor, DP—with prior images acquired from different vendor).

Mammograms used in test sets in this study were full-field digital mammograms (FFDM) taken from the BreastScreen Australia image bank. Four senior radiologists (W.L, H.F, G.L and N.B), who were designated lead radiologists for BreastScreen and had more than 25 years of experience in interpreting screening mammograms, curated the nine test sets. Each set consisted of 60 bilateral mammograms, making up a total of 540 mammographic images (361 normal and 179 cancer). Among those, 245 cases (69 cancer and 176 normal) had prior images acquired from the same vendor and 129 cases (47 cancer and 82 normal) had prior images from a different vendor (Figure 1). In each test set, cases with and without prior images were displayed in random order. The vendors of images included General Electric, Sectra, Hologic, Fujifilm and Siemens HealthCare. Prior images were obtained from the previous screening round (approximately 2 years) before the current images used for the test sets. There were 166 cases without prior images (63 cancer and 103 normal) which were mammograms taken from the first screening of patients. Cancer cases were biopsy-proven whilst normal cases were confirmed by at least two BreastScreen radiologists after a negative follow up report conducted two years post the current mammography.

Each case consisted of two-view mammograms, a cranio-caudal (CC) and mediolateral-oblique (MLO), of the left and right breast. The senior curating radiologists evaluated the quality of all the mammograms and pathology reports prior to defining the location and the size of the lesions on the mammograms as ground truth. These experts also confirmed the breast density level of each case using the BI-RADS system as well as the cancer types for those cases that contained an abnormal lesion (classified as masses, architectural distortion, calcification, asymmetric density and mix of types). The distribution of low (A-almost entirely fatty and B-scattered fibroglandular density) and high (C-heterogeneously dense and D-extremely dense) groups of breast density were equal at 50%. Spiculated mass and discrete mass were the most common types of cancer lesions within the test sets (49.2%) and 54% of lesions had a size of equal to or less than 10mm. The participants remained unaware of the number of abnormal lesions in each set, although they were informed that these mammogram sets were enriched with cancer cases. The features of mammogram collection were described in Table 2. 

### 2.4. Mammogram Display and Reading Environments

Radiologists conducted the reading of mammographic test sets on at least 5 MP monitor workstations dedicated for viewing mammograms, which had a maximum display luminance of 600 cd/m^2^ and an ambient lighting of no more than 30 lux. If prior images were not available, the current images were displayed in 1-row × 4-column mode with the order of views being RMLO-LMLO-RCC-LCC. If prior images were available for a case, it was first presented in 2-row × 4-column display mode in which the current mammograms were shown on the upper row and the prior mammograms were displayed on the lower row. Readers could use the keypad to switch to full-screen display of each mammogram view or select the hanging to compare images. Higher spatial-resolution display of a section of a mammogram was available to the readers via applying the full-screen mode or using an electronic magnifying glass.

### 2.5. Mammogram Reading Procedure

In each mammogram set, readers were asked to find and localize abnormal lesions (if there were any) on mammograms using a lexicon from two to five with the following classifications: 2—possible benign lesions; 3—indeterminate/equivocal finding; 4—suspicious of malignancy; and 5—highly suggestive of malignancy. If the reader could not detect any lesions on an image, the case was automatically recorded as score 1 (normal). Readers could backtrack and adjust their decisions at any time before clicking the “submit” button. The BREAST platform was used to record the diagnostic decision of each reader for each case [10,12]. Information about the clinical experience of readers was obtained via an electronic questionnaire embedded into the platform prior to the commencement of reading sessions. 

The performances of the radiologists were evaluated by comparing their report for each case and the ground truth. The rating each radiologist assigned for each case was based on rates 1 and 2 being considered as normal cases, while rates 3, 4 and 5 were recognized as abnormal cases. A lesion was considered as localized correctly when the distance from the mathematical centre of the lesion, that a reader marked to the centre of true cancer location, was equal to or less than radius of the true cancer lesion. Readers were free to report multiple lesion locations and only the highest rating was used for data analysis. 

### 2.6. Statistical Analysis

True positive (correct cancer case detection), true negative (correct normal case reporting) and recall rates of radiologists were calculated separately for cases without prior images (NP), cases with prior images from the same vendor as current images (SP), and cases with prior images from a different vendor (DP)). AUC (Area under the ROC Curve) values which measured the ability of radiologists to distinguish cancer and normal cases were compared among three case groups using the DeLong method [13]. Logistic regression was used to estimate the odds ratio (OR) of true positive (TP), true negative (TN) and true cancer lesion localization (TL) in each comparison for all cases and for different levels of breast density and lesion characteristics. The Pearson’s chi-squared test was performed to compare TP, TN, TL in different cohorts. The diagnostic accuracy of radiologists with different experience (number of cases reading per week and number of years in reading mammograms) were also investigated. *p* < 0.05 indicates a significant statistical result. Statistical analyses were conducted using SPSS software (version 23, Chicago, IL, USA).

## 3. Results

### 3.1. Radiologists’ Performances among Cases without Prior, with Prior Images from Same Vendor and Different Vendor in Cancer-Enriched Test Sets

The recall rates were higher in the NP (0.444) and DP (0.461) cases than in the SP cases (0.353). True positive rates in NP and DP cases were 12.8% and 10.3% higher than SP cases (0.803 and 0.785 vs. 0.712). In contrast, true negative rates in NP and DP cases were 4.8% and 2.0% lower than SP cases (0.749 and 0.771 vs. 0.787). The AUC values for NP and DP cases were significantly higher than that for SP cases (0.814 and 0.820 vs. 0.782; *p* < 0.0001). Higher AUC values for NP and DP cases compared with SP cases were also found in both low breast density cases (0.819 vs. 0.800; <0.05) and high breast density cases (0.810 and 0.818 vs. 0.759; *p* < 0.0001) (Table 3).

### 3.2. Comparison of Odds Ratio of True Positive and True Negative for Three Groups of Cases

Radiologists were more likely to detect cancer cases in NP than SP cases (OR = 1.64; 95%CI:1.49–1.81; *p* < 0.0001) and the odds ratio of true positive for DP relative to SP cases was 1.48 (95%CI:1.33–1.64; *p* < 0.0001). With true negative, the odds ratio for NP relative to SP cases was 0.81 (95%CI:0.76–0.86; *p* < 0.0001), DP relative to SP cases was 0.91 (95%CI:0.84–0.99; *p* = 0.023), NP relative to DP cases was 0.89 (95%CI:0.82–0.97; *p* = 0.008). 

In term of breast density, a similar significant finding was found with the odds ratio of true positive for NP relative to SP cases being 1.52 (low density) and 1.86 (high density) (*p* < 0.0001), DP relative to SP cases being 1.21 (*p* = 0.011) (low density) and 1.88 (*p* < 0.0001) (high density). With regard to the true negative, the odds ratio for NP relative to SP cases was 0.74 (*p* < 0.0001), DP relative to SP cases was 0.84 (*p* = 0.004), NP relative to DP cases was 0.87 (*p* = 0.031) in low breast density cases, while in high breast density cases, odds ratio was only significantly for NP relative to SP cases (OR = 0.88; *p* = 0.006) (Table 4).

### 3.3. Comparison of Odds Ratio of True Cancer Lesion Location for Three Groups of Cases

The odds ratios of true cancer localization in NP and DP relative to SP cases for architectural distortion were 1.75 and 3.23 (*p* < 0.0001), for calcifications it was 2.85 with the OR of DP relative to SP cases (*p* < 0.0001), while the odds ratios for NP relative to DP cases were 0.54 and 0.41 (*p* < 0.0001), respectively, for these two types of lesions. With masses, the odds ratios of NP relative to SP and DP cases were 1.34 and 1.32 (*p* < 0.05) for discrete masses, and 1.98 and 1.53 (*p* < 0.0001) for spiculated masses. The odds ratios of true cancer localization in NP and DP relative to SP cases for a lesion ≤10 mm were 1.1 and 1.2 (*p* < 0.05); for a lesion >10 mm, they were 1.25 and 1.75 (*p* < 0.05) (Table 5).

### 3.4. Performances of Radiologists with Different Levels of Working Experience

**Figure 2:**
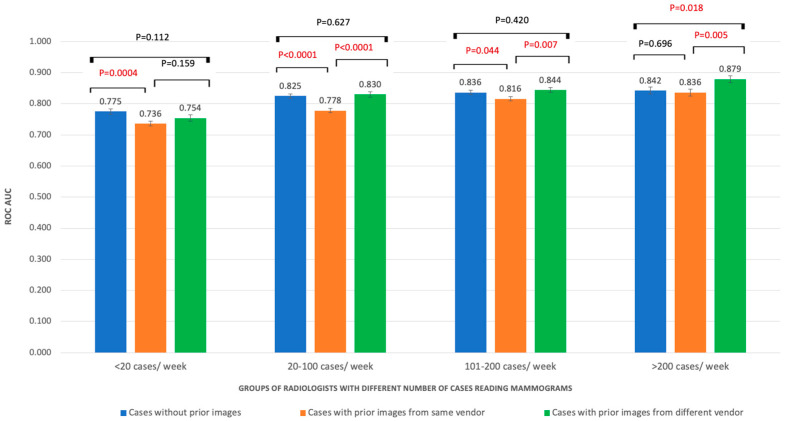
ROC AUC performances of radiologists with different experience in number of cases read per week.

**Figure 3:**
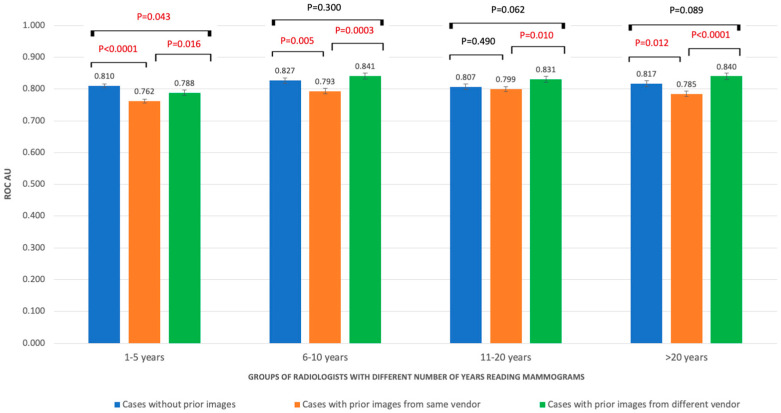
ROC AUC performances of radiologists with different experience in number of years reading mammograms.

The AUC values of the three groups of cases were improved with the increase in the number of mammograms that radiologists were reading per week. Significantly higher AUC in NP than SP cases was found in radiologists reading less than 20 mammograms per week (0.78 vs. 0.74; *p* = 0.0004). When the reading volume was increased, the AUC in DP and NP cases were higher than the AUC of SP cases (*p* < 0.05). With radiologists reading more than 200 cases per week, the AUC value of DP (0.88) was significantly higher than NP (0.842; *p* = 0.018) and SP cases (0.0836; *p* = 0.005) (Figure 2). Similarly, radiologists in most groups of years’ reading mammograms obtained significant higher AUC values in NP and DP cases than SP cases (*p* < 0.05) (Figure 3).

## 4. Discussion

In general, results from this study showed that radiologists obtained higher true positive rates and AUC values when reading cases without prior images, or with prior images obtained from different vendors, than cases with prior mammograms undertaken from the same vendor. The study also provided an estimation of the odds ratio for true positive, true negative and true cancer lesion localization among three case groups in different levels of breast density and lesion characteristics.

The practice of comparison between present and previous/prior mammograms is a strategy that is often transferred through generations of radiologists via supervisor to trainee sessions. Radiology is a visual specialty and the appreciation of progression or regression of the disease process, and specifically to mammography parenchymal density and architectural changes, may often be appreciated through previous, comparable radiographs. However, the benefit of viewing the prior images in the domain of screening mammography has only really been debated in the era of FS technology. In the study of Thurfjell et al. (2000), 150 FS mammograms with and without prior images, including 35 cancer cases, were examined by three radiologists and all readers significantly increased their specificity when prior mammograms were available for comparison; however, the effect on sensitivity was unclear as it showed that the rate of cancers detected by radiologists was reduced from 40.3% to 37.7%, when reading the mammograms with prior images [6]. In another study, Burnside et al. (2002) retrospectively analysed results from 48,281 FS mammograms and compared detection rates between cases with and without prior mammograms [14]. The authors found that comparison with previous examinations decreased the recall rate from 4.9% to 3.8%; however, this did not significantly affect the cancer detection rate (5.5 vs. 5.2/1000; *p* = 0.87).

Later, in 2007, Roelofs et al. [15] explored the value of prior cases using 160 digitized FS mammograms (80 cancers), evaluated by 12 radiologists in two reading modes: firstly without prior images and after 4 weeks, with priors available if a recall value of 3 or above was assigned. There was no significant difference in the number of localized lesions between the two reading modes and authors suggested that prior mammograms seemed not play an important role in the initial detection of abnormalities. A recent monitoring report of the BreastScreen program by the Australian Institute of Health and Welfare [16] showed that the sensitivity of 2-year screening was 83.9% for the first screening round and 76.5% for subsequent screening rounds, which might relate partly to the opportunity to review prior mammograms. Unfortunately, the previous studies did not include the information if the previous mammograms were taken from the same or a different vendor, and there have been no recent studies which have used FFDM for both the prior and current cases.

Compared with previous studies, our study involved a large number of radiologists (612) and we also used FFDM rather than FS mammograms, with a total of 540 mammograms (361 normal and 179 cancer) of which 374 cases had prior images (245 cases with prior images from the same vendor and 129 from different vendor). It also explored the impact of breast density, different lesion types, lesion sizes, as well as work experience on the diagnostic efficacy of radiologists in reading mammograms with and without prior images. The results showed that the identification of normal cases was significantly better when prior mammograms were available, especially if the images were taken from the same vendor, for comparison with current images. In regard to cancer detection rate, radiologists performed better in cases without prior images and cases featured with previous mammograms recorded by a different manufacturer than cases with prior images from the same vendor across a different level of breast density. This may shed new light on the previously unanswered questions from FS studies [5,14], whereby sensitivity may be improved with FFDM via its capacities to digitally alter the images to seek clarification on difficult areas or regions of interest, which is also supported by the higher recall rates for NP and DP cases than for the SP cases. Digital radiography systems, created by various manufacturers, might use different image processing algorithms to align the histogram values with the look-up-table translation curve. This may cause the image grayscale and corresponding image to look different among mammography machine vendors, and the difference between mammograms taken from different systems might have an impact on the diagnostic performances of radiologists. From this, we hypothesize that ‘different vendor’ prior cases may offer additional visual information which is not available from the same vendor’s prior images. Interestingly, radiologists scored higher sensitivity for the cases that had no prior images (NP) than for the DP cases with low breast density. This might relate to satisfaction-of-reporting bias which refers to the tendency to perpetuate the interpretation of a prior imaging study [17]. In a screening scenario, reviewing prior images with the majority of cases having normal low density breast may lead a radiologist to overestimate the probability of a normal interpretation and small abnormal findings could be missed; this pitfall is also known as an alliterative error [17,18]. Satisfaction-of-reporting bias has been attributed to the tendency and/or need of people in a social or professional group to conform to the views of their peers. Therefore, a suggested reading protocol could be that radiologists should review the examination findings and generate an impression before reading the prior images. Our results, generalized from an educational setting to the clinical setting, suggest that prior mammograms taken from the same vendor might be best deployed after review of the current images to assist with resolving ambiguity regarding possible malignancy, but may be less helpful to review at the same time as initial decision making.

This study also reported that the ROC AUC value, which measures the ability of radiologists to distinguish cancer and normal cases in three groups of cases, increased in accordance with the increase in the reader experience considering the variety of different reading volumes per week and number of years of experience. The minimum annual volume of mammographic readings is often used as a desirable level of experience and this minimum value can vary across countries such as 2000 readings per year in Australia and Canada [19,20] and 5000 readings in the United Kingdom [21]. In Australia, evidence from previous studies [19,22] demonstrated that radiologists with more than 2000 readings annually outperformed those with less than 1000 readings. The results from our study signal that the confidence of experienced readers to downgrade suspicious or indeterminate interpretations to normal or benign improved in combination with increased experience in reading mammograms. This can suggest that radiologists with the highest reading volume and highest years of experience were able to detect cancer and identify normal cases better than readers with low reading volume [3]. Therefore, the use of prior image types could be considered based on the experience of radiologists to enhance their diagnostic accuracy, as most radiologists performed better with DP and NP cases compared with SP cases, especially in specific types of lesions such as architectural distortion, calcification and spiculated mass.

Although the empirical design of this study allowed us to explore differences in the performances of radiologists in reading mammograms with and without prior images, we cannot observe the impact of other rounds of previous mammograms as our study only used one round of prior imaging, which was taken closest to the current mammograms (approximately 2-year interval). It is understood that the time for a lesion to evolve to a more suspicious lesion depends on its nature and on the interval between current and priors. In a previous study, Hayward et al. (2016) found that comparison with two or more prior mammograms resulted in a significant reduction in the recall rate (10%) and increased in the cancer detection rate relative to comparison with a single prior mammogram (2.3 cases/1000 examinations) [23]. Further work is also needed to fully understand the precise mechanism behind the findings, and why prior images from the same vendor were not always useful for radiologists in detecting cancers. Such research might include eye-tracking technology to track how radiologists view current and prior mammograms. Another limitation of our study is that it was conducted in the experimental training setting with cancer-enriched test sets that both might influence the sensitivity and specificity of radiologists; although, previous research has reported a considerable level of agreement between BREAST test-set performances and clinical audit [24]. Hayward et al.’s study found that reading cases without prior mammograms resulted in a 10% increase in the recall rate of screening mammograms compared with reading cases with at least a single prior mammogram (16.6% vs. 6.3%) [23]. In our study, although the recall rates of three groups of cases (between 0.35 and 0.46) were significantly higher than in the screening environment, due to our usage of cancer-enriched mammogram sets, the difference in recall rates between cases with (0.39) and without prior images (0.44) is 6%, which is in line with the previous study. Finally, there was a limited number of cases in different lesion types in our study. Although a high number of readers included in our study could compensate for this limitation to some extent, further investigation with a larger number of lesion sub-types will be needed to confirm the findings. 

## 5. Conclusions

The study showed that cases without previous mammograms or with prior mammograms obtained from different vendors were more likely to benefit radiologists in cancer detection than cases with prior mammograms obtained from the same vendor. The findings suggest exploring a mixed reading strategy in viewing cases with prior mammograms acquired from the same and different manufacturers to enhance the diagnostic accuracy of breast cancer detection in the digital era.

## Figures and Tables

**Table 1:** Radiologists’ characteristics.

Categories		Number of Readers (%)
**Number of mammograms read per week**	<20	185 (30.2%)
	20–60	112 (18.3%)
	61–100	73 (11.9%)
	101–150	84 (13.7%)
	151–200	90 (14.7%)
	>200	68 (11.2%)
**Number of years reading mammograms**	≤5	168 (27.5%)
	6–10	181 (29.6%)
	11–15	72 (11.8%)
	16–20	71 (11.6%)
	>20	120 (19.5%)

**Table 2:** Mammogram characteristics.

		Cases with No Prior Images	Cases with Prior Images from Same Vendor	Cases with Prior Images from Different Vendor	Total Cases (%)
**Normal cases**	Breast density A	7	15	16	38 (10.5%)
	Breast density B	38	73	33	144 (39.9%)
	Breast density C	44	65	26	135 (37.4%)
	Breast density D	14	23	7	44 (12.2%)
	*Total*	*103*	*176*	*82*	*361*
**Cancer cases**	Breast density A	4	9	5	18 (10.1%)
	Breast density B	21	33	22	76 (42.5%)
	Breast density C	30	19	17	66 (36.9%)
	Breast density D	8	8	3	19 (10.6%)
	*Total*	*63*	*69*	*47*	*179*
**Lesion types**	Architectural distortion	6	4	5	15 (8.4%)
	Asymmetric density	7	7	7	21 (11.7%)
	Calcifications	12	10	8	30 (16.8%)
	Discrete mass	10	11	13	34 (19%)
	Stellate/Spiculated mass	18	22	13	53 (29.6%)
	Mix of types	9	14	3	26 (14.5%)
	*Total*	*49*	*57*	*37*	*143*
**Lesion sizes**	≤10 mm	35	35	27	97 (54.2%)
	>10 mm	28	34	20	82 (45.8%)
	*Total*	*63*	*69*	*47*	*179*

**Table 3:** Diagnostic performances of radiologists among cases without prior, and with prior images from same vendor and different vendor.

		NP vs. SP	DP vs. SP	NP vs. DP
**All cases**	Recall rate	0.444–0.353	0.461–0.353	0.444–0.461
	True positive rate	0.803–0.712	0.785–0.712	0.803–0.785
	True negative rate	0.749–0.787	0.771–0.787	0.749–0.771
	ROC AUC	0.814–0.782; *p* < 0.0001 *	0.82–0.782; *p* < 0.0001 *	0.814–0.82; *p* = 0.349
**Cases with low breast density (A–B)**	Recall rate	0.430–0.365	0.436–0.365	0.430–0.436
	True positive rate	0.806–0.733	0.768–0.733	0.806–0.768
	True negative rate	0.754–0.806	0.779–0.806	0.754–0.779
	ROC AUC	0.819–0.800; *p* = 0.0172 *	0.819–0.800; *p* = 0.0207 *	0.819–0.819; *p* = 0.97
**Cases with high breast density (C–D)**	Recall rate	0.454–0.340	0.491–0.340	0.454–0.491
	True positive rate	0.800–0.683	0.803–0.683	0.800–0.803
	True negative rate	0.746–0.769	0.761–0.769	0.746–0.761
	ROC AUC	0.810–0.759; *p* < 0.0001 *	0.818–0.759; *p* < 0.0001 *	0.810–0.818; *p* = 0.364

Notes: *: *p* < 0.05. NP: Cases with no prior images, SP: Cases with prior images from same vendor, DP: Cases with prior images from different vendor.

**Table 4:** Estimated Odds Ratio (OR) of true positive and true negative of mammograms in comparison of cases without prior images, with prior images from the same vendor and with prior images from different vendor.

		Comparison	OR (95% CI)	*p*-Value
**True positive**		NP vs. SP	1.643 (1.489–1.812)	<0.0001 *
	All cases	DP vs. SP	1.478 (1.33–1.643)	<0.0001 *
		NP vs. DP	1.111 (0.991–1.246)	0.07
		NP vs. SP	1.517 (1.306–1.762)	<0.0001 *
	Cases with low breast density (A–B)	DP vs. SP	1.205 (1.043–1.393)	0.011 *
		NP vs. DP	1.259 (1.06–1.494)	0.008 *
		NP vs. SP	1.859 (1.626–2.126)	<0.0001 *
	Cases with high breast density (C–D)	DP vs. SP	1.884 (1.613–2.202)	<0.0001 *
		NP vs. DP	0.987 (0.843–1.154)	0.866
**True negative**		NP vs. SP	0.808 (0.756–0.864)	<0.0001 *
	All cases	DP vs. SP	0.909 (0.837–0.987)	0.023 *
		NP vs. DP	0.889 (0.815–0.971)	0.008 *
		NP vs. SP	0.736 (0.665–0.815)	<0.0001 *
	Cases with low breast density (A–B)	DP vs. SP	0.844 (0.752–0.948)	0.004 *
		NP vs. DP	0.872 (0.769–0.988)	0.031 *
		NP vs. SP	0.884 (0.808–0.966)	0.006
	Cases with high breast density (C–D)	DP vs. SP	0.958 (0.85–1.079)	0.481
		NP vs. DP	0.922 (0.815–1.044)	0.199

Notes: *: *p* < 0.05. NP: Cases with no prior images, SP: Cases with prior images from same vendor, DP: Cases with prior images from different vendor.

**Table 5:** Relative Odds Ratio (OR) of true positive lesion localization on mammograms in a comparison of cases without prior images, with prior images from the same vendor and with prior images from a different vendor.

Lesion Characteristics	Comparison	OR (95% CI)	*p*-Value
Architectural distortion	NP vs. SP	1.746 (1.313–2.322)	<0.0001 *
	DP vs. SP	3.232 (2.37–4.408)	<0.0001 *
	NP vs. DP	0.54 (0.417–0.7)	<0.0001 *
Calcification	NP vs. SP	1.158 (0.942–1.423)	0.163
	DP vs. SP	2.854 (2.223–3.664)	<0.0001 *
	NP vs. DP	0.406 (0.317–0.52)	<0.0001 *
Discrete mass	NP vs. SP	1.343 (1.073–1.68)	0.01 *
	DP vs. SP	1.016 (0.813–1.27)	0.89
	NP vs. DP	1.322 (1.038–1.683)	0.023 *
Asymmetric density	NP vs. SP	1.03 (0.819–1.297)	0.798
	DP vs. SP	1.187 (0.922–1.527)	0.183
	NP vs. DP	0.868 (0.68–1.109)	0.257
Stellate/Spiculated mass	NP vs. SP	1.984 (1.671–2.355)	<0.0001 *
	DP vs. SP	1.297 (1.09–1.544)	0.003 *
	NP vs. DP	1.529 (1.257–1.861)	<0.0001 *
Lesion ≤ 10 mm	NP vs. SP	1.115 (1.011–1.23)	0.029 *
	DP vs. SP	1.18 (1.058–1.316)	0.003 *
	NP vs. DP	0.945 (0.846–1.056)	0.317
Lesion > 10 mm	NP vs. SP	1.254 (1.04–1.512)	0.018 *
	DP vs. SP	1.748 (1.498–2.041)	<0.0001 *
	NP vs. DP	0.564 (0.448–0.711)	<0.0001 *

Notes: *: *p* < 0.05. NP: Cases with no prior images, SP: Cases with prior images from same vendor, DP: Cases with prior images from different vendor.

## Data Availability

The data that support the findings of this study are available upon reasonable request. The data are not publicly available due to privacy or ethical restrictions.

## Abbreviations

Film-screen (FS), full-field digital mammography (FFDM), cases without prior images (NP), cases with prior images obtained from the same vendor (SP), cases with prior images obtained from different vendor (DP), ROC AUC (Receiver Operating Characteristic Area Under the Curve).
